# Do Health Technology Assessment organisations consider manufacturers’ costs in relation to drug price? A study of reimbursement reports

**DOI:** 10.1186/s12962-022-00383-y

**Published:** 2022-08-31

**Authors:** Joost J. Enzing, Saskia Knies, Jop Engel, Maarten J. IJzerman, Beate Sander, Rick Vreman, Bert Boer, Werner B. F. Brouwer

**Affiliations:** 1 Erasmus School of Health Policy & Management, Erasmus University Rotterdam, Rotterdam, The Netherlands; 2 Zorginstituut Nederland, Diemen, The Netherlands; 3 Cancer Health Services Research, School of Population and Global Health, Faculty of Medicine, Dentistry and Health Sciences, University of Melbourne, Melbourne, VIC Australia; 4 Health Technology and Services Research Department, Faculty of Behavioural, Management and Social Sciences, Technical Medical Centre, University of Twente, Enschede, The Netherlands; 5 Toronto Health Economics and Technology Assessment (THETA) Collaborative, University Health Network, Toronto, ON Canada; 6 Institute of Health Policy, Management and Evaluation (IHPME), University of Toronto, Toronto, ON Canada; 7 Public Health Ontario, Toronto, ON Canada; 8 ICES, Toronto, ON Canada; 9 Division of Pharmacoepidemiology and Clinical Pharmacology, Utrecht Institute for Pharmaceutical Sciences, Utrecht University, Utrecht, The Netherlands

**Keywords:** Health Technology Assessment, HTA, HTA agencies, Reimbursement decision making, Profitability, Profit margin, Drug pricing, Pharmaceuticals, Pricing models

## Abstract

**Introduction:**

Drug reimbursement decisions are often made based on a price set by the manufacturer. In some cases, this price leads to public and scientific debates about whether its level can be justified in relation to its costs, including those related to research and development (R&D) and manufacturing. Such considerations could enter the decision process in collectively financed health care systems. This paper investigates whether manufacturers’ costs in relation to drug prices, or profit margins, are explicitly mentioned and considered by health technology assessment (HTA) organisations.

**Method:**

An analysis of reimbursement reports for cancer drugs was performed. All relevant Dutch HTA-reports, published between 2017 and 2019, were selected and matched with HTA-reports from three other jurisdictions (England, Canada, Australia). Information was extracted. Additionally, reimbursement reports for three cases of expensive non-oncolytic orphan drugs prominent in pricing debates in the Netherlands were investigated in depth to examine consideration of profit margins.

**Results:**

A total of 66 HTA-reports concerning 15 cancer drugs were included. None of these reports contained information on manufacturer’s costs or profit margins. Some reports contained general considerations of the HTA organisation which related prices to manufacturers’ costs: six contained a statement on the lack of price setting transparency, one mentioned recouping R&D costs as a potential argument to justify a high price. For the case studies, 21 HTA-reports were selected. One contained a cost-based price justification provided by the manufacturer. None of the other reports contained information on manufacturer’s costs or profit margins. Six reports contained a discussion about lack of transparency. Reports from two jurisdictions contained invitations to justify high prices by demonstrating high costs.

**Conclusion:**

Despite the attention given to manufacturers’ costs in relation to price in public debates and in the literature, this issue does not seem to get explicit systematic consideration in the reimbursement reports of expensive drugs.

**Supplementary Information:**

The online version contains supplementary material available at 10.1186/s12962-022-00383-y.

## Introduction

In collectively financed health systems reimbursement decisions regarding new pharmaceuticals, in a number of jurisdictions, are informed by health technology assessment reports and the result of systematic decision processes. Such reimbursement decisions regarding pharmaceuticals are often made based on a price set by the manufacturer of the drug. This price typically covers all costs relevant to the manufacturer as well as a profit margin. Often, the relative sizes of these components of the final (list) price are unclear. In some cases, the price a manufacturer sets for its product may be considered high (in absolute sense or given its effects), which can lead to public and scientific debates about whether this price is justified [1, 2]. Such debates are fuelled by the growth in new, highly priced drugs (and other technologies for that matter), leading to questions about the sustainability of current pricing and reimbursement models [3]. However, whether manufacturers’ costs (including those for research and development (R&D), manufacturing, marketing and overheads) in relation to price, and therefore their profit margin, are available to HTA organisations and are explicitly considered by these organisations in current reimbursement decisions concerning drugs, to our knowledge, has not been examined.

In the context of reimbursement decisions it is important to distinguish between the assessment and the appraisal phase of the decision-making process. All available evidence, mostly clinical and economic, is collected and synthesised during the assessment phase. It is unlikely that information on manufacturers’ costs or profits will be disclosed at the time of submission and assessment for reasons of confidentiality. Indeed, despite the increased attention for price setting, manufacturers’ costs and profit margins are not part of the common assessment criteria considered during the HTA process. For example, in the EUnetHTA Core Model [4], a European HTA framework, neither manufacturers’ costs nor profit margins, are mentioned. These topics are also normally not covered in overviews of used or proposed decision criteria [5–7]. However, despite not being available at the assessment phase, the information may still be relevant in the appraisal phase supporting the decision-making process. In the appraisal phase, typically, a committee critically appraises the available scientific evidence but also can consider societal and ethical aspects deemed relevant in reaching a decision or making a recommendation. Prices and profit margins could be discussed and weighed in this phase, alongside other broader considerations regarding the evaluated technology, in reaching a final decision or recommendation. Moreover, the increased reliance on price negotiations in the reimbursement process (e.g. [8]) may suggest that decision makers expect that there at least *could* be room for price reductions, which in turn may suggest the expectation that it would be possible to negotiate towards an acceptable profit margin. Such negotiations can also be part of or the result of the appraisal phase. Hence, given the increased attention for price setting as well as the increased reliance on price negotiations, manufacturers’ costs in relation to prices, or profit margins, could be an explicit part of the deliberations during the appraisal phase, also to justify certain decisions or recommendations. A recent discrete choice experiment among Dutch healthcare decision makers suggested that information on profit margin would influence their reimbursement recommendations when available [9].

Whether and how manufacturers’ costs and profits are currently addressed in the appraisal phase of reimbursement decisions, is an important but understudied topic. How appraisal committees consider this issue may also be related to their views on price setting and the context in which a new intervention will be used. Regarding the latter, the need for active attention for and negotiations of prices may be affected by the competitiveness of the market a technology enters into after a reimbursement decision. Regarding the former, views on ‘desirable’ price setting range from value-based approaches, relating prices more to added (therapeutic) value than to the costs of manufacturing the product [10] to cost-based approaches that take the manufacturers’ costs as a starting point for determining ‘reasonable’ or ‘fair’ prices [11]. Implicit or explicit negotiations could help to achieve such ‘reasonable’ prices, also determining profit margins. In so, they determine the division of the generated surplus, i.e., the monetary difference between manufacturers’ costs and maximum willingness to pay, between the manufacturer and society. Their success, however, will also depend on relative negotiating power [12].

Considerations of manufacturers’ costs in relation to price and (expected) profit margins may be relevant for appraisal committees in formulating a decision or advice in relation to a specific reimbursement decision. Given the attention for and potential relevance of profits for reimbursement decisions in different contexts, this study therefore investigates, for selected jurisdictions, whether manufacturers’ costs in relation to price are currently explicitly considered by HTA organisations as reflected in reimbursement reports of expensive drugs. In doing so, it is acknowledged that the phases of assessment, appraisal and price negotiations may be organised differently in different jurisdictions and not always be fully distinguishable. Such reports are publicly available and provide insight into the explicit deliberations of appraisal committees and the arguments used in this context. To our knowledge, this study is the first to address this issue. Although not all deliberations may be documented within these reports, the results may contribute to our understanding of the role of manufacturers’ profits in current reimbursement decisions.

## Methods

A study of HTA-reports, documents, or sets of documents reporting a reimbursement decision, was performed to investigate whether manufacturers’ costs in relation to prices were explicitly considered by the HTA organisation. This study consisted of both an analysis of systematically selected cancer drugs reports and three case studies on expensive non-oncolytic orphan drugs. For pragmatic reasons, the study was limited to reports published in English or Dutch. To cover a wide geographical range but still a manageable amount of documents, HTA-organisations from four jurisdictions were selected, namely Zorginstituut Nederland (ZIN; the Netherlands), the National Institute for Health and Care Excellence (NICE; England), the Canadian Agency for Drugs and Technologies in Health (CADTH; Canada), and the Pharmaceutical Benefit Advisory Committee (PBAC) and the Medical Services Advisory Committee (MSAC) (Australia). HTA-reports were obtained from the respective websites in June 2020. These reports generally contain results from both the assessment and the appraisal phase.

The analysis of systematically selected reports was limited to decisions concerning cancer drugs, as these pharmaceuticals are generally expensive, and discussions about manufacturers’ cost in relation to price may be expected to be relatively prominent for such products. As a starting point, relevant reports from ZIN, published between 2017 and 2019, were selected from the ZIN website, restricted to those containing the keyword oncology. The resulting reports were screened independently by two reviewers (JJE and JE) and included when these considered a cancer drug (excluding e.g. diagnostics). For the cancer drugs which were the subject of the included ZIN HTA-reports, corresponding HTA-reports in the other three jurisdictions were retrieved for the considered cancer drug and indication, accepting minor differences in indication or drug combinations. This approach facilitated comparison across the four jurisdictions. For the Australian jurisdiction, where resubmissions are common, inclusion of oncology reports was limited to first submissions.

The analysis of cancer drug reports was supplemented with three in depth case studies of expensive non-oncolytic orphan drugs because of their prominence in the pricing debate in the Netherlands: lumacaftor/ivacaftor (Orkambi^®^) for the treatment of cystic fibrosis, eculizumab (Soliris^®^) for the treatment of paroxysmal nocturnal haemoglobinuria (PNH) and eculizumab (Soliris^®^) for the treatment of atypical haemolytic uraemic syndrome (aHUS). These cases, which represent ‘extremes’ in proposed prices, were purposely selected to increase the change of observing a discussion on price in relation to manufacturers’ costs. Available HTA-reports on these products were retrieved for all four jurisdictions and investigated in terms of their discussion of prices in relation to costs, similarly to those on cancer drugs.

The collected reports were read independently by two reviewers (JJE and JE) who extracted data using a structured data extraction form implemented in Microsoft Excel (Microsoft, Redmond, WA). To provide insight in general characteristics of the assessed drugs and the reimbursement recommendations, additional information on cost-effectiveness, budget impact and price negotiations for the included products was collected, as well as information on the final reimbursement decision (JE, validated by JJE).

Firstly, information on the manufacturers’ costs or the manufacturers’ profits related to the evaluated drug was extracted. For example, this extraction would include the mentioning of specific investments needed for the development of the drug. During this extraction the reviewers used the following broad definitions:

- Manufacturers’ costs include past, present and future costs related to the product and borne by the manufacturer.
- Manufacturers’ profits (or profit margins) are financial benefits for the manufacturer realized when revenues generated by the drug exceed the costs to the manufacturer.

Secondly, the reviewers extracted text fragments which contained considerations on manufacturers’ costs in relation to price. Signal words used during this extraction were: price, costs, R&D, manufacturing, overhead, profits, profit margin, substantiation, fairness, fair, reward for innovation, recouping and transparency. This extraction would, for example, include discussions on the potential role of manufacturers’ costs within the reimbursement decision process. Considerations relating prices to cost-effectiveness or budget impact, which may also contain the used signal words, were excluded as these considerations concern costs to the payer, and not costs to the manufacturer. Considerations solely present within external stakeholder comments included in the reports, without explicit reflection by the HTA organisation, were not included, as these were not interpreted as considerations of the HTA organisation. However, considerations presented by an HTA organisation as a result of their reviews were included. The reviewers combined their extraction results, and in case of disagreement, this was resolved by discussion with two additional authors (SK and RV).

## Results

In this section we will describe the results of our analysis of cancer drugs reports and subsequently of our orphan drugs case studies.

### Analysis of HTA-reports on cancer drugs

**Fig. 1:**
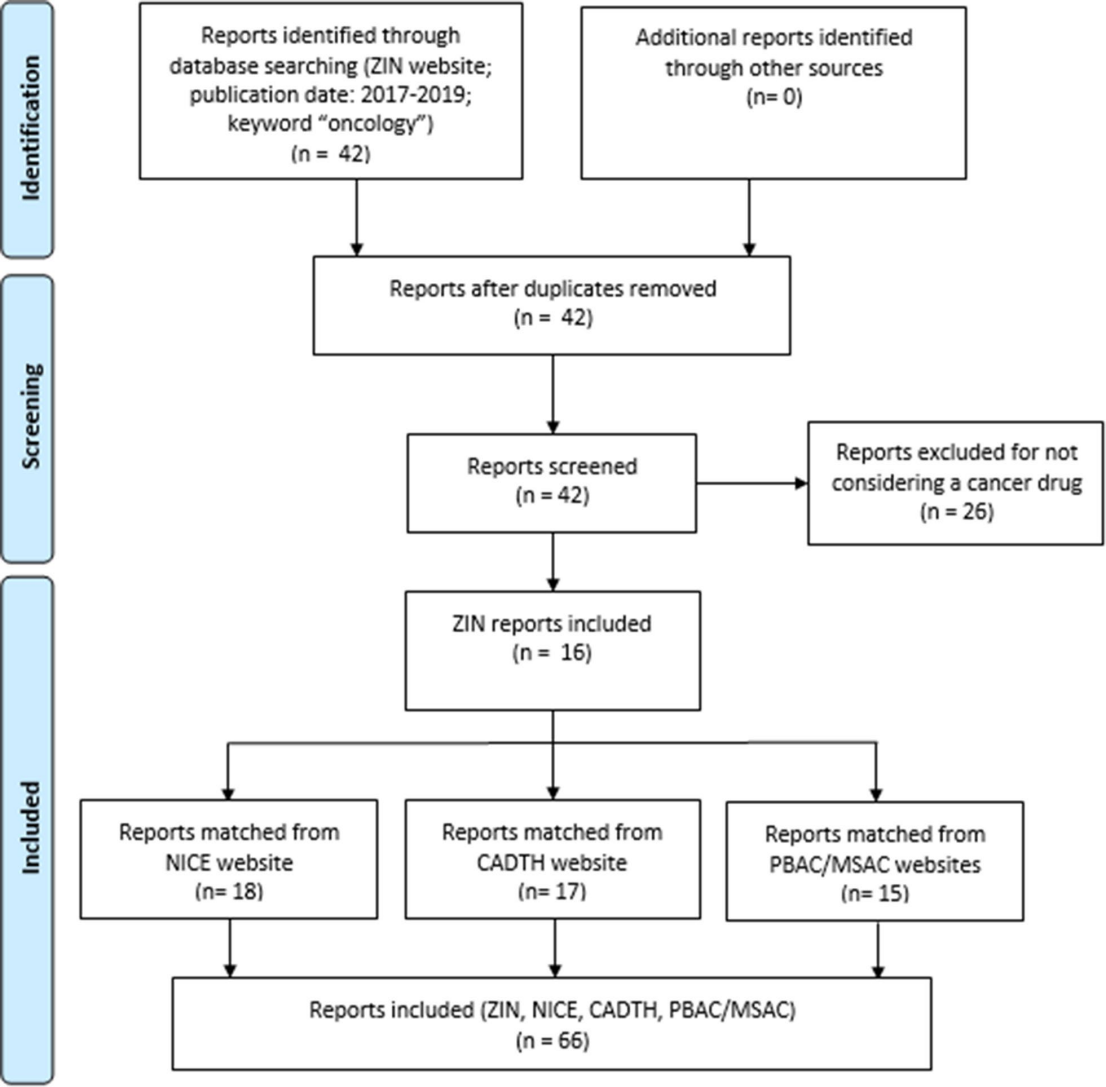
PRISMA flow diagram: inclusion of cancer drug reports

Of all relevant reports published by ZIN in the years 2017, 2018 and 2019 (n = 42), 16 HTA reports of a cancer drug were included in the analysis (see Table 1). From the websites of the other HTA-organisations, 18 NICE reports, 17 CADTH reports and 15 PBAC/MSAC reports were retrieved. Some of the Dutch reports considered two indications for the same drug, while these indications were reported in separate documents by NICE and/or CADTH, which explains the higher number of included reports from these two institutions. Overall, 66 reports were included (see Additional file 1: Table S1 for references; see Fig. 1 for a PRISMA Flow Diagram).

**Table 1:** Included cancer drugs in the analysis and number of reports per jurisdiction

Active ingredient /generic name	Brand name	Indication	ZIN	NICE	CADTH	PBAC/MSAC
Dabrafenib and trametinib	Tafinlar and mekenist	Melanoma	1	1	1	1
Ipilimumab and nivolumab	Yervoy and Opdivo	Renal cell carcinoma	1	1	1	1
Venetoclax and retuximab	Venclyxto and generic	Chronic lymphocytic leukaemia	1	1	1	1
Durvalumab	Imfinzi	Non-small cell lung cancer	1	1	1	1
Abemaciclib	Verzenios	Breast cancer	1	2	1	1
Axicabtagene ciloleucel	Yeskarta	Large B-cell lymphoma	1	1	1	1
Tisagenlecleucel	Kymriah	Large B-cell lymphoma/Acute lymphocytic leukaemia	2	2	1	2
Dinutuximab beta	Qarziba	Neuroblastoma	1	1	1	0
Osimertinib	Tagrisso	Non-small cell lung cancer	1	1	1	1
Atezolizumab	Tecentriq	Non-small cell lung cancer	1	1	1	1
Ribociclib	Kisqali	Breast cancer	1	1	1	1
Daratumumab	Darzalex	Myeloma	1	1	1	1
Cetuximab	Erbitux	Colorectal cancer	1	1	1	1
Ibrutinib	Imbruvica	Lymphocytic leukaemia	1	1	2	1
Palbociclib	Ibrance	Breast cancer	1	2	2	1

*ZIN* Zorginstituut Nederland, *NICE* National Institute for Health and Care Excellence, *CADTH* Canadian Agency for Drugs and Technologies in Health, *PBAC* Pharmaceutical Benefit scheme and medical services Advisory, *MSAC* Medical Services Advisory Committee

None of the 66 reports contained information on manufacturers’ costs or profit margins of the evaluated drugs. Seven reports contained a consideration which related manufacturers’ costs to price: six discussed a perceived lack of pricing transparency and one mentioned recouping development costs.

Widely used criteria in the context of reimbursement recommendations were found to be (the uncertainty around the) effectiveness, cost-effectiveness and budget impact. Often, reports included conditions under which final reimbursement could be allowed, including price reductions. Within the investigated reports, references to price negotiations were common: 10 out of 16 ZIN reports recommended to start price negotiations, 16 out of 18 NICE reports mentioned a negotiated discount, 16 out of 17 CADTH reports recommended price negotiations and 11 out of 15 PBAC/MSAC reports mentioned (proposed) special pricing arrangements.

#### The Netherlands

None of the 16 ZIN reports contained explicit information about manufacturers’ costs or profit margins of the evaluated drugs. Four reports (daratumumab; palbociclib; ribociclib; atezolizumab) contained a statement regarding a perceived lack of transparency regarding price setting by the manufacturer, which was part of the concluding recommendations. It was not stated whether or how transparency was sought by the committee. One other report (venetoclax and rituximab) indicated that price negotiations were recommended, also because an increase in volume was expected and development costs could be recouped more quickly.

Four reports contained a positive recommendation for reimbursement without requiring further price negotiations. Two reports contained a negative advice, without recommending further price negotiations. The other ten reports recommended to start price negotiations (four reports) or indicated that price reductions would be a condition for reimbursement (six reports). The recommendations to start price negotiations were typically substantiated with arguments regarding uncertainty concerning effectiveness, an unfavourable incremental cost-effectiveness ratio (ICER) and/or a high budget impact.

#### England

None of the 18 NICE reports contained information on manufacturers’ costs or profit margins of the evaluated drugs. Moreover, none of the 18 reports contained considerations relating manufacturers’ costs to prices, as put forward by NICE or the manufacturer.

One appraisal resulted in a negative recommendation (osimertinib), while the other appraisals resulted in recommending the product (nine for routine use, eight for use within the context of the cancer drug fund). The positive recommendations were made under the condition that confidential commercial agreements were followed, which explicitly included providing a negotiated discount in 16 reports.

#### Canada

None of the 17 CADTH reports contained information on manufacturers’ costs or profit margins of the evaluated drugs. In an appendix of the ethics and implementation report of tisagenlecleucel, CADTH cited two references which both pointed to a lack of transparency in pricing of CAR T-cell therapies [13, 14]. In one of these, De Lima and colleagues [13] stated that there is a ‘*need for greater transparency about pricing given public investment into R&D*’. These two references [13, 14] were also present in the ethics review of axicabtagene ciloleucel, which additionally cited a recommendation of the Association of European Cancer Leagues to better explain the rationale behind the prices of CAR T-cell therapies [15]. No other considerations relating manufacturers’ costs to price were found within the CADTH reports.

Of the reports, one resulted in not recommending the product (cetuximab), all others resulted in a conditional positive advice given to the provinces. The relevant conditions in all cases involved making price-arrangements with the manufacturer and improving cost-effectiveness.

#### Australia

None of the 15 PBAC/MSAC reports, in which some data may be censored during redaction, contained information on manufacturers’ costs or profit margins of the evaluated drugs, or any considerations which related manufacturers’ costs to price.

Five reports recommended reimbursement in combination with some form of (price) arrangements. Ten reports either deferred making a decision (two reports) or did not recommend the reimbursement (eight reports) of the appraised drug based on the first submission. Rejections were accompanied by an invitation to resubmit the application, which is common practice in Australia, as can be illustrated by a sentence used in all included PBAC reports (e.g., osimertinib): “*A PBAC decision not to recommend listing or not to recommend changing a listing does not represent a final PBAC view about the merits of the medicine. A company can resubmit to the PBAC or seek independent review of the PBAC decision.*” It should be noted that drugs rejected for Pharmaceutical Benefits Scheme (PBS) listing may be available through self-funding or private insurance. Resubmissions may involve an adjusted price, and in that sense may be viewed as being part of a negotiation process with the HTA organisation. All reports contained a reference to (proposed) special pricing arrangements (11 reports), or to a required price reduction.

### Orphan drugs case studies

From the websites of the four relevant HTA-organisations, 21 reports were identified concerning the (re)appraisal of eculizumab for PNH, eculizumab for aHus or lumacaftor/ivacaftor for the treatment of cystic fibrosis. One document reported cost related arguments provided by the manufacturer to substantiate the relatively high price of the product (eculizumab aHus, NICE). This document, as well as five others (eculizumab PNH, ZIN, 2016; eculizumab PNH, ZIN, 2017; eculizumab aHus, ZIN, 2016; lumacaftor/ivacaftor, ZIN, May 2016; lumacaftor/ivacaftor, ZIN, December 2016), discussed manufacturers’ costs as relevant information for the reimbursement decision (Table 2) (see Additional file 1: Table S2 for references).

**Table 2:** Included HTA-reports related to orphan drugs case studies

Intervention (indication)	HTA organisation	Publication date	Consideration of manufacturers’ cost in relation to drug price?
Eculizumab (PNH)	PBAC	July 2008	No
	PBAC	March 2009	No
	CADTH	February 2010	No
	PBAC	July 2010	No
	ZIN	May 2016	Yes
	ZIN	June 2017	Yes
Eculizumab (aHus)	PBAC	March 2013	No
	CADTH	July 2013	No
	PBAC	March 2014	No
	PBAC	August 2014	No
	NICE	January 2015	Yes
	CADTH	May 2015	No
	ZIN	November 2016	Yes
Lumacaftor/ivacaftor (CF)	PBAC	March 2016	No
	ZIN	May 2016	Yes
	NICE	July 2016	No
	CADTH	October 2016	No
	PBAC	November 2016	No
	ZIN	December 2016	Yes
	PBAC	July 2017	No
	PBAC	July 2018	No

*HTA*  health technology assessment, *PBAC*  Pharmaceutical Benefit scheme and medical services Advisory Committee, *CADTH* Canadian Agency for Drugs and Technologies in Health, *ZIN* Zorginstituut Nederland, *NICE* National Institute for Health and Care Excellence, *PNH*  paroxysmal nocturnal haemoglobinuria, *aHus*  atypical haemolytic uraemic syndrome, *CF * cystic fibrosis

#### Eculizumab for treatment of PNH

The orphan drug eculizumab is used to treat patients with paroxysmal nocturnal haemoglobinuria (PNH). PNH is a life-threatening genetic disease which results in severe medical complications such as anaemia and thrombosis. In May 2016 ZIN published an HTA-report on eculizumab for PNH. This report concluded that eculizumab, at annual costs of € 360,000 per patient, was not cost-effective and ZIN recommended to suspend reimbursement and start price negotiations. Much weight was attached to the notion that the manufacturer was not transparent about the price structure. The appraisal committee indicated to take the position that rejection of reimbursement would be in order ‘*if the manufacturer does not take the trouble to submit an acceptable cost-effectiveness model and asks an extremely high price that, according to the ACP, is not transparent as well as being immoral*…’.

The report also mentioned that for interventions with an unfavourable cost-effectiveness, arguments may exist to justify reimbursement. Such justifications could include costs related to the development of the drug, to market access, and to production. Furthermore, a general call for transparency was included, directed to the pharmaceutical industry. This should help in making accountable public decisions regarding reimbursement. In addition, the Minister of Health (MoH) was advised to take into consideration, in the context of the process of price negotiations, that other indications for which eculizumab would be prescribed were expected. In a reassessment in 2017, ZIN reported that the requested transparency was still lacking.

No NICE report on eculizumab for the indication of PNH was found.

CADTH published a common drug review on eculizumab for PNH in 2010. In this report the Canadian Drug Expert Committee (CDEC) recommended that eculizumab should not be listed at the price listed in the submission. The yearly cost of eculizumab per patient were labelled as exceptionally high. The CDEC noted that eculizumab had not been shown to be cost-effective, with the remark that this criterion is weighed against other criteria in making reimbursement decisions. The CDEC added: ‘*It has been argued that the costs of drugs to treat rare diseases are often high because of the relatively small number of patients for whom the drug is indicated*.’ This sentence could imply that actual costs might be used to justify high prices, even if these would result in estimates beyond commonly used cost-effectiveness thresholds.

PBAC published a report on eculizumab for PNH in 2008. The PBAC rejected the manufacturers’ submission on the basis of unacceptably high and highly uncertain costs per avoided death. Reassessment followed in 2009 when eculizumab was rejected on the basis of an unacceptably high and highly uncertain ICER. In 2010, the drug was appraised in the context of the Life Saving Drugs Program (LSDP), which provides access to expensive and potentially lifesaving drugs for very rare life-threatening conditions. In this appraisal the PBAC decided to defer the submission for eculizumab to allow the sponsor time to obtain further data about the magnitude of the survival gain. Since January 2011 eculizumab is funded through the LSDP. None of the PBAC reports mentioned manufacturers’ costs or profits.

#### Eculizumab for treatment of aHus

Eculizumab is also indicated for the treatment of patients with atypical haemolytic uraemic syndrome (aHUS). Like PNH, aHus is a life-threatening genetic orphan disease, resulting in anaemia, thrombocytopenia and kidney failure. In November 2016 ZIN published their HTA-report on eculizumab for the treatment of aHus. ZIN concluded that eculizumab, at annual costs of € 478,000 per patient, was not cost-effective and recommended to only allow reimbursement under strict conditions, including price negotiations. In their deliberations, ZIN considered that the manufacturer, although requested to do so, did not offer transparency to explain or justify the high price. Moreover, the manufacturer did not provide a sound estimate of cost-effectiveness. Similar to their eculizumab PNH reports, ZIN acknowledged that insight provided by manufacturers into their costs could potentially justify reimbursing an intervention with an unfavourable ICER.

In January 2015 NICE published their HTA-report on eculizumab for the treatment of aHus. Based on its high price, even when compared to the prices of other highly specialised technologies, NICE asked the manufacturer to provide a price justification. The response by the manufacturer, which highlighted the need to recoup development costs in a small number of patients, was deemed insufficient. An additional inquiry was made by NICE, specifically aimed at exceptional clinical or safety requirements during clinical development, costs of post-marketing research plans, and any other information to justify the proposed price. The manufacturer provided a response in which they stated that R&D costs accounted for only a small part of the additional costs for highly specialised drugs. Other elements mentioned in this context included the need to set up multiple sites for patient recruitment into clinical trials, investments in education, higher risk of failure, and reinvestment for new indications. This response did not convince NICE, as these aspects were not considered to be exclusively valid for eculizumab. Moreover, the number of patients treated with the drug was found not to be lower than that of some other highly specialised drugs. In their response, the manufacturer expressed concern that the committee was acting outside its remit with NICE’s inquiries pointing towards a more cost-based price substantiation. However, the committee stated that it is within their remit to ’… *also take into account what could be considered a reasonable cost for the medicine in the context of recouping manufacturing, research and development costs from sales to a limited number of patients.*’

CADTH recommended in 2013 that eculizumab should not be listed since its clinical benefit could not be adequately established. In 2015 it confirmed this recommendation while adding the need to consider opportunity costs and healthcare system sustainability given the associated “very high cost per patient”. Both reports did not contain information on manufacturers’ costs or profit margins of the evaluated drug or any considerations relating price to manufacturers’ costs.

In 2013 PBAC rejected eculizumab for the treatment of aHus on the basis of uncertainty regarding clinical effectiveness and an unacceptably highICER. Later (March 2014; August 2014) reassessments were published which concluded that eculizumab could be accepted through special arrangements, including a scheme of outcome-based price rebates. No comments on manufacturers’ costs or profit margins were found within these reports.

#### Lumacaftor/ivacaftor for the treatment of cystic fibrosis

Lumacaftor/ivacaftor is an orphan drug used to treat patients with the inherited disease cystic fibrosis. Cystic fibrosis causes severe effects on the lungs and the digestive system of patients. In May 2016 ZIN published an initial HTA-report concerning lumacaftor/ivacaftor which concluded that it was not cost-effective. ZIN stated that acceptance of an ICER above the common threshold could be possible in cases where price setting would be transparent, but that such transparency was lacking for lumacaftor/ivacaftor. They explicitly called for more transparency regarding the price setting of lumacaftor/ivacaftor. This was also deemed important since the ICER of the drug to a considerable degree was influenced by its price (resulting in annual costs of € 169.386 per patient) and was well above the relevant threshold. In a reassessment published 7 months later ZIN concluded that the requested transparency was still not provided. Eventually, lumacaftor/ivacaftor was accepted for reimbursement after (confidential) price negotiations.

NICE published an HTA-report in July 2016 which concluded that the estimated ICERs for lumacaftor/ivacaftor were considerably higher than what is normally considered a cost-effective use of NHS resources. Similar to ZIN, NICE did not recommend reimbursement of lumacaftor/ivacaftor. Transparency regarding price setting was not discussed within the report. In 2019, NHS England announced a commercial agreement with the manufacturer that would allow access to the drug.

In 2016 CADTH also did not recommend reimbursement of lumacaftor/ivacaftor in a common drug review. This recommendation was justified by a lack of proven effectiveness. No comments on manufacturers’ costs or profit margins were made.

The PBAC decided not to recommend lumacaftor/ivacaftor for Pharmaceutical Benefits Scheme (PBS) listing based on the uncertain and unfavourable ICER and the uncertain long-term effectiveness. No reference was made to manufacturers’ costs or profit margins (March 2016). These were also not considered in the context of three subsequent resubmissions (November 2016; 2017; 2018). After their 2018 meeting, PBAC recommended lumacaftor/ivacaftor to be listed on the PBS, making it available via a Managed Access Program (MAP).

## Discussion

This study aimed to investigate whether manufacturers’ costs in relation to prices, i.e., profit margins, are explicitly considered by HTA organisations within their reimbursement reports. A total of 66 HTA-reports on cancer drugs were studied to investigate whether information on manufacturers’ costs or profit margin of the evaluated drug were included, and to see whether general considerations were included which relate manufacturers’ costs to proposed prices. In addition, three extreme cases in the area of highly expensive orphan drugs were studied. In total, 21 HTA-reports on these three pharmaceutical products were investigated. In general, information on manufacturers’ costs and profit margins of the evaluated drugs was not presented in the reports. Only one report contained (non-quantitative) information on manufacturers’ costs (eculizumab aHus, NICE, 2015). This information was provided by the manufacturer as part of a price justification. The justification however did not convince the appraisal committee. In 13 of the 87 reports, general considerations relating manufacturers’ costs to prices were provided by the HTA organisation, mostly in the form of statements on the (undesirable) lack of transparency on price setting. Requests for more transparency were not honoured.

The results indicate that information on manufacturers’ costs in relation to prices is typically lacking and typically does not seem to be requested. Reflections of HTA-organisations on the relationship between manufacturers’ costs and prices are rare and, if present, typically very general. At the same time, the instrument of price negotiations was recommended and used, explicitly or implicitly through resubmissions, quite common. This appears to signal the more general idea that proposed prices (and hence implied profits) could be lowered by manufacturers.

This study was the first, to our knowledge, to investigate this topic. That also implies that we cannot relate our results to previous findings in the literature. However, our findings do appear to align with a recent review of methodological guidelines of 24 HTA-organisations, including the four jurisdictions we investigated. Manufacturers’ costs nor profit margins were reported as criteria used in priority setting [16].

### Limitations

We acknowledge several limitations of our study. First, this study is based on publicly available, partly censored, reports, which may not report all deliberations of the involved committees during their meetings, especially in jurisdictions which limit their publication to a summary.

Second, our study was focused on cancer drugs as well as purposely selected cases of orphan drugs. This sample of reports may not be representative for reports in general. One might expect considerations related to manufacturers’ costs in relation to price to be relatively frequently present in our sample, which would make our findings in terms of attention for this topic (albeit rare) an overestimation of the attention for this issue in general.

Third, although we found little explicit attention for manufacturers’ costs in relation to prices and profitability, we cannot exclude the possibility that this attention was present more implicitly through the more common criteria of cost-effectiveness and budget impact. For example, advising price reductions or negotiations, based on an unfavourable cost-effectiveness will (ceteris paribus) lead to lower profit rates. Moreover, when a high budget impact was used to provide a cue for price negotiations, this may be framed as a consideration of affordability, but could also relate to the assumption that fixed manufacturers’ cost are recouped after a certain overall revenue. A related example of this type of influence is NICE’s policy to use a higher threshold for some orphan drugs than for non-orphan drugs [17]. This may relate to the limited number of patients available to recoup investment costs. In that sense, manufacturers’ costs in relation to prices may play a larger role than observed. Such implicit considerations may also be enforced by the fact that decision frameworks typically do not use profitability (in some form) as criterion. Our study focused only on explicit information and consideration of this issue, which therefore is an important limitation.

Fourth, we had few reports with final unconditional negative decisions in our sample. In such cases one might expect profitability to more frequently play a role in justifying such a decision. This could be an interesting avenue for future research.

When interpreting our findings across jurisdictions it should be acknowledged that differences in reporting may complicate comparisons. For example, PBAC only publishes summaries of their appraisals, while in other jurisdictions more extensive HTA-reports are published. Additionally, important differences exist in the process of appraisal in relation to price negotiations, which also hamper international comparisons in this context. For example, in England, price negotiations may take place during the assessment and appraisal process. Therefore, part of the NICE HTA-reports were able to already take into account the final negotiation results (e.g. a lowered price, Managed Entry Agreements). It could be argued that in these cases, a public consideration of manufacturers’ costs in relation to the proposed or negotiated price may not be that relevant or even desirable (also for the HTA agency). Similarly, in Australia, negotiations may take the form of resubmissions with a reduced price offer, allowing ‘negotiation outcomes’ through this process to be part of the final appraisal, although not necessarily of the preceding reports. In Canada and the Netherlands such negotiations follow after an HTA-report has been published and serve as input for the negotiations. Hence, for instance in a number of Dutch HTA-reports, subsequent price negotiations are recommended and the required price reduction sometimes is even quantified [18]. In such a context, the emphasis on manufacturers’ costs in relation to the proposed price might be expected to be larger. Additionally, the emphasis on profitability during reimbursement decision making may depend on pricing policies in place within a specific jurisdiction. In England for example, the growth of the medicines budget is capped by 2% per annum through an agreement between the pharmaceutical industry and the government (the ‘Voluntary scheme for branded medicines pricing and access’). As a so-called portfolio-wide profit control scheme, this agreement may limit the need for considerations on profitability at the individual product level. The use of external reference pricing, in place for pharmaceuticals in for example the Netherlands, may also limit this need.

### Interpretation and implications of the results

The investigated HTA-reports typically did not contain any information on manufacturers’ costs or on the profit margin of the assessed drug. As a consequence, recommendations, including those concerning the start of a price negotiation, were not substantiated with an explicit weighing of manufacturers’ costs or profit margins. In other words, the reports lacked the information required to take an informed, cost-based, or partly cost-based, approach to the appraisal. In a limited number of reports we found indications that such a partly cost-based approach implicitly is used in considering the desirability of reimbursing a particular drug. While such considerations may play a larger role in practice through the adopted processes, the use of price negotiations or through the use of other criteria (e.g. cost-effectiveness, budget impact), at least in the studied HTA-reports these considerations were not systematically mentioned.

To emphasize the way in which these issues may still enter the debate and HTA-reports, we highlight the NICE dinutuximab beta appraisal consultation document. This document contained public comments which featured manufacturers’ costs and profit margins (NICE, 2018). In that document, a carer urged the manufacturer ‘*to be as transparent as possible in laying out the basis on which it has developed its pricing to show it is offering the drug as cheaply as possible while meeting its obligations to shareholders*’. An NHS professional stated that ‘… *developing a new drug for an orphan indication such as high risk NB is never going to be profitable for a pharmaceutical company, particularly a relatively small one like EusaPharma unless it is priced above what NICE would normally consider cost-effective*’. Another NHS professional commented ‘… *drug development is never cheap. The costs can be recouped with relatively narrow profit margin if a drug has a market of tens of thousands of patients. If companies are squeezed too hard, then they will be disincentivised from researching drugs for rare conditions* …’. NICE replied that it noted these comments. While it is not clear how NICE considered them, they do highlight that HTA-organisations likely are aware of such considerations, even if they are not explicitly elaborated on in their reports.

In a general sense therefore, perceived manufacturers’ costs in relation to proposed prices may at least play an implicit role, for instance in the decision to recommend to start price negotiations. However, this was not explicitly stated in the reports, which in general substantiate the need for price negotiations by pointing to insufficient cost-effectiveness. Invitations to manufacturers to provide a cost-based pricing substantiation to justify initially asked prices were observed in some cases, especially when the proposed price resulted in an ICER exceeding the relevant threshold. This again highlights the relevance of profitability, but also may suggest that exceeding common ICER thresholds could be considered acceptable for products with a limited profit margin.

While in the current situation price negotiations are typically advised based on common HTA criteria such as cost-effectiveness, policy makers could explore the desirability of starting price negotiations based on an expected large profit margin, also when the ICER does not exceed the threshold. This was not observed in any of the HTA-reports in our study. Starting price negotiations for cost-effective products with high profit margins could meet some concerns relating to high prices. Obviously, such decisions would normally be informed by information that is currently lacking: exact cost information. Moreover, in deciding on starting a price negotiation other aspects which influence expected negotiation outcomes, e.g. market position, patents and available alternative products, would also be relevant.

New price models that could guide such price negotiations have been proposed in the literature, all with their own advantages and disadvantages. Some of these models explicitly distinguish between manufacturers’ costs and profits in price setting, also in evaluating a new drug. For example, Berdud et al. describe a method to establish a “reasonable” price for orphan drugs based on the costs of conducting research and the size of the patient population [19]. Balderrama et al. propose a model in which a drug price can be labelled “justifiable” or “unjustifiable” by considering the costs of its development and manufacturing [20]. Uyl et al. [21] propose to estimate reasonable prices for new cancer drugs by estimating manufacturers’ total average unit costs for a pharmaceutical to which a relevant and acceptable profit margin could then be added. In their approach, this profit margin could be based on the anticipated clinical benefit, leading to a more intermediate position between fully cost-based and value-based prices. As a final example, van den Berg et al. [22] present a cost-based approach to calculate a fair price, specifically for a repurposed orphan drug.

Within the context of high drug prices, the public contributions to drug development are a topic of interest in the literature [23]. Public funding of drug R&D is shown to be substantial in specific cases e.g. [24], and it is claimed that one to two-thirds of total upfront R&D costs are funded by taxpayers or charitable donations [25]. When manufacturers’ costs are related to drug price, this public funding should be acknowledged and transparency should be provided, also to prevent governments to “pay twice”.

If it is considered desirable to broaden the HTA process to more systematically and explicitly consider profitability, actively requiring information on manufacturers’ costs seems necessary. In that context it is interesting to mention a recent French law which requires pharmaceutical companies to disclose the amounts of public investments in R&D for specific new drugs entering their market [26]. This information is then allowed to be used by the responsible government body (Comité économique des produits de santé, CEPS) during price negotiations with pharmaceutical companies. Note that legal amendments to enforce more extensive disclosures about manufacturing costs, for example on costs of active ingredients and profits, were not approved by the French parliament, emphasising the difficulty in obtaining and using such information in practice.

## Conclusion

Despite the attention given to manufacturers’ costs in relation to price within the literature and in public debates, and although they appear to have been considered relevant in some decisions, profitability levels do not seem to receive systematic explicit attention in HTA-reports for expensive drugs.

## Supplementary Information

**Additional file 1: Table S1.** References to the HTA-reports on cancer drugs included in the analysis (n = 66). **Table S2.** References to the included HTA-reports related to the cases on orphan pharmaceuticals (n = 21).

## Data Availability

The data collected and analyzed during the study are available from the corresponding author on reasonable request.
