# Supplementary material for: Do Health Technology Assessment organisations consider manufacturers’ costs in relation to drug price? A study of reimbursement reports
Source: Cost Eff Resour Alloc. 2022 Aug 31;20:46. doi: 10.1186/s12962-022-00383-y (PMC9434877; doi:10.1186/s12962-022-00383-y)
Supplement: Supplementary file 1 — Additional file 1: Table S1. References to the HTA-reports on cancer drugs included in the analysis (n = 66). Table S2. References to the included HTA-reports related to the cases on orphan pharmaceuticals (n = 21). [file 12962_2022_383_MOESM1_ESM.docx]

**Additional information**

**S1 Table. References to the HTA-reports on cancer drugs included in the analysis (n=66)**

| **Active ingredient /generic name** | **HTA organisation** | **Publication year** | **Title, URL** |
| --- | --- | --- | --- |
| abemaciclib | CADTH | 2019 | "Abemaciclib for advanced or metastatic Breast Cancer (PC0161-000)", https://www.cadth.ca/abemaciclib-advanced-or-metastatic-breast-cancer-details |
| abemaciclib | NICE | 2019 | "Abemaciclib with an aromatase inhibitor for previously untreated, hormone receptor-positive, HER2-negative, locally advanced or metastatic breast cancer - Technology appraisal guidance", www.nice.org.uk/guidance/ta563 |
| abemaciclib | NICE | 2019 | "Abemaciclib with fulvestrant for treating hormone receptor-positive, HER2-negative advanced breast cancer after endocrine therapy - Technology appraisal guidance", www.nice.org.uk/guidance/ta579 |
| abemaciclib | PBAC | 2019 | "Public Summary Document – March 2019 PBAC Meeting", https://www.pbs.gov.au/industry/listing/elements/pbac-meetings/psd/2019-03/files/abemaciclib-psd-march-2019.pdf |
| abemaciclib | ZIN | 2019 | "Pakketadvies abemaciclib (Verzenios®)", https://www.zorginstituutnederland.nl/publicaties/adviezen/2019/03/07/pakketadvies-sluisgeneesmiddel-abemaciclib-verzenios |
| atezolizumab | CADTH | 2018 | "Tecentriq for Non-Small Cell Lung Cancer (PC0115-000)", https://www.cadth.ca/tecentriq-non-small-cell-lung-cancer-details |
| atezolizumab | NICE | 2018 | "Atezolizumab for treating locally advanced or metastatic non-small-cell lung cancer after chemotherapy - Technology appraisal guidance", www.nice.org.uk/guidance/ta520 |
| atezolizumab | PBAC | 2017 | "Public Summary Document – November 2017 PBAC Meeting ", https://www.pbs.gov.au/industry/listing/elements/pbac-meetings/psd/2017-11/files/atezolizumab-psd-november-2017.pdf |
| atezolizumab | ZIN | 2018 | [“Pakketadvies atezolizumab (Tecentriq®)”, https://www.zorginstituutnederland.nl/publicaties/adviezen/2018/02/07/pakketadvies-atezolizumab-tecentriq-bij-gemetastaseerde-niet-kleincellige-longkanker](https://www.zorginstituutnederland.nl/publicaties/adviezen/2018/02/07/pakketadvies-atezolizumab-tecentriq-bij-gemetastaseerde-niet-kleincellige-longkanker) |
| axicabtagene ciloleucel | CADTH | 2019 | "Axicabtagene Ciloleucel for Adults With Relapsed or Refractory Large B-cell Lymphoma", https://www.cadth.ca/axicabtagene-ciloleucel-adults-relapsed-or-refractory-large-b-cell-lymphoma |
| axicabtagene ciloleucel | NICE | 2019 | "Axicabtagene ciloleucel for treating diffuse large B-cell lymphoma and primary mediastinal large B-cell lymphoma after 2 or more systemic therapies - Technology appraisal guidance", www.nice.org.uk/guidance/ta559 |
| axicabtagene ciloleucel | MSAC | 2020 | "Public Summary Document Application No. 1587 – Axicabtagene ciloleucel (CAR-T therapy) for the treatment of refractory or relapsed CD19-positive lymphoma", http://www.msac.gov.au/internet/msac/publishing.nsf/Content/1587-public |
| axicabtagene ciloleucel | ZIN | 2019 | "Pakketadvies axicabtagene ciloleucel (Yescarta®)", https://www.zorginstituutnederland.nl/publicaties/adviezen/2019/03/07/pakketadvies-sluisgeneesmiddel-axicabtagene-ciloleucel-yescarta |
| cetuximab | CADTH | 2014 | "Erbitux for Metastatic Colorectal Cancer (PC0031-000)", https://www.cadth.ca/erbitux-metastatic-colorectal-cancer-details |
| cetuximab | NICE | 2017 | "Cetuximab and panitumumab for previously untreated metastatic colorectal cancer - Technology appraisal guidance", www.nice.org.uk/guidance/ta439 |
| cetuximab | PBAC | 2014 | "Public Summary Document – November 2014 PBAC Meeting", https://www.pbs.gov.au/industry/listing/elements/pbac-meetings/psd/2014-11/files/cetuximab-psd-11-2014.pdf |
| cetuximab | ZIN | 2017 | "Standpunt cetuximab (Erbitux®) bij gemetastaseerd coloncarcinoom (herbeoordeling)", https://www.zorginstituutnederland.nl/publicaties/standpunten/2017/08/10/cetuximab-erbitux-bij-gemetastaseerd-coloncarcinoom-herbeoordeling |
| dabrafenib & trametinib | CADTH | 2019 | "Tafinlar & Mekinist in combo Melanoma Adjuvant Therapy (PC0152-000)", https://www.cadth.ca/tafinlar-mekinist-combo-melanoma-adjuvant-therapy-details |
| dabrafenib & trametinib | NICE | 2018 | "Dabrafenib with trametinib for adjuvant treatment of resected BRAF V600 mutation-positive melanoma - Technology appraisal guidance", www.nice.org.uk/guidance/ta544 |
| dabrafenib & trametinib | PBAC | 2019 | "Public Summary Document – March 2019 PBAC Meeting", https://www.pbs.gov.au/industry/listing/elements/pbac-meetings/psd/2019-03/files/dabrafenib-and-trametinib-psd-march-2019.pdf |
| dabrafenib & trametinib | ZIN | 2019 | "Pakketadvies dabrafenib/trametinib (Tafinlar®/Mekinist®)", https://www.zorginstituutnederland.nl/publicaties/adviezen/2019/08/28/pakketadvies-sluisgeneesmiddelen-dabrafenib-in-combinatie-met-trametinib-tafinlar-en-mekinist-bij-de-adjuvante-behandeling-van-volwassen-patienten-met-stadium-iii-melanoom |
| daratumumab | CADTH | 2017 | "Darzalex for Multiple Myeloma (second-line or beyond) (PC0104-000)", https://www.cadth.ca/darzalex-multiple-myeloma-second-line-or-beyond-details |
| daratumumab | NICE | 2019 | "Daratumumab with bortezomib and dexamethasone for previously treated multiple myeloma - Technology appraisal guidance", www.nice.org.uk/guidance/ta573 |
| daratumumab | PBAC | 2017 | "Public Summary Document – November 2017 PBAC Meeting", https://www.pbs.gov.au/industry/listing/elements/pbac-meetings/psd/2017-11/files/daratumumab-psd-november-2017.pdf |
| daratumumab | ZIN | 2017 | [“Pakketadvies daratumumab (Darzalex)”, https://www.zorginstituutnederland.nl/publicaties/adviezen/2017/11/gvs-advies-oordruppels-bij-gehoorgangontsteking-otitis-externa/pakketadvies-daratumumab-darzalex-bij-multipel-myeloom](https://www.zorginstituutnederland.nl/publicaties/adviezen/2017/11/gvs-advies-oordruppels-bij-gehoorgangontsteking-otitis-externa/pakketadvies-daratumumab-darzalex-bij-multipel-myeloom) |
| dinutuximab | CADTH | 2019 | "Unituxin for Neuroblastoma (PC0154-000)", https://www.cadth.ca/unituxin-neuroblastoma-details |
| dinutuximab beta | NICE | 2018 | “Dinutuximab beta for treating high-risk neuroblastoma [ID910] – Single technology appraisal”, https://www.nice.org.uk/guidance/ta538/documents/committee-papers-2 |
| dinutuximab beta | ZIN | 2018 | "Standpunt dinutuximab bèta (Qarziba®) bij de behandeling van hoog-risico neuroblastoom bij patiënten van 12 maanden en ouder", https://www.zorginstituutnederland.nl/publicaties/standpunten/2018/12/12/standpunt-dinutuximab-beta-qarziba-bij-de-behandeling-van-hoog-risico-neuroblastoom-bij-patienten-van-12-maanden-en-ouder |
| durvalumab | CADTH | 2019 | "Imfinzi for Non-Small Cell Lung Cancer (PC0131-000)", https://www.cadth.ca/imfinzi-non-small-cell-lung-cancer-details |
| durvalumab | NICE | 2019 | "Durvalumab for treating locally advanced unresectable non-small-cell lung cancer after platinum-based chemoradiation - Technology appraisal guidance", www.nice.org.uk/guidance/ta578 |
| durvalumab | PBAC | 2018 | "Public Summary Document – November 2018 PBAC meeting", https://www.pbs.gov.au/industry/listing/elements/pbac-meetings/psd/2018-11/files/durvalumab-psd-november-2018.pdf |
| durvalumab | ZIN | 2019 | "Pakketadvies durvalumab (Imfinzi®)", https://www.zorginstituutnederland.nl/publicaties/adviezen/2019/04/01/pakketadvies-sluisgeneesmiddel-durvalumab-imfinzi-voor-volwassenen-met-lokaal-gevorderd-irresectabel-niet-kleincellig-longcarcinoom |
| ibrutinib | CADTH | 2015 | "Imbruvica for Chronic Lymphocytic Leukemia/Small Lymphocytic Lymphoma (previously treated) (PC0043-000)", https://www.cadth.ca/imbruvica-chronic-lymphocytic-leukemiasmall-lymphocytic-lymphoma-previously-treated-details |
| ibrutinib | CADTH | 2016 | "Imbruvica for Waldenström’s Macroglobulinemia (PC0082-000)", https://www.cadth.ca/imbruvica-waldenstroms-macroglobulinemia-details |
| ibrutinib | NICE | 2017 | "Ibrutinib for previously treated chronic lymphocytic leukaemia and untreated chronic lymphocytic leukaemia with 17p deletion or TP53 mutation - Technology appraisal guidance", www.nice.org.uk/guidance/ta429 |
| ibrutinib | PBAC | 2017 | "Public Summary Document – November 2017 PBAC Meeting ", https://www.pbs.gov.au/industry/listing/elements/pbac-meetings/psd/2017-11/files/ibrutinib-mcl-psd-november-2017.pdf |
| ibrutinib | ZIN | 2017 | "Pakketadvies ibrutinib (Imbruvica®)", https://www.zorginstituutnederland.nl/publicaties/adviezen/2017/06/08/pakketadvies-ibrutinib-imbruvica-bij-de-eerstelijnsbehandeling-chronische-lymfatische-leukemie-cll-bij-patienten-die-geen-del17p-of-tp53-mutatie-hebben |
| ipilimumab & nivolumab | CADTH | 2018 | "Opdivo in combo with Yervoy for Renal Cell Carcinoma (PC0132-000)", https://www.cadth.ca/opdivo-combo-yervoy-renal-cell-carcinoma-details |
| ipilimumab & nivolumab | NICE | 2019 | "Nivolumab with ipilimumab for untreated advanced renal cell carcinoma - Technology appraisal guidance", www.nice.org.uk/guidance/ta581 |
| ipilimumab & nivolumab | PBAC | 2018 | "Public Summary Document – July 2018 PBAC Meeting", https://www.pbs.gov.au/industry/listing/elements/pbac-meetings/psd/2018-07/files/nivolumab-and-ipilimumab-melanoma-psd-july-2018.pdf |
| ipilimumab & nivolumab | ZIN | 2019 | "Pakketadvies ipilimumab/nivolumab (Yervoy®/Opdivo®)", https://www.zorginstituutnederland.nl/publicaties/adviezen/2019/05/29/pakketadvies-sluisgeneesmiddelen-ipilimumab-yervoy-in-combinatie-met-nivolumab-opdivo |
| osimertinib | CADTH | 2019 | "Tagrisso for Non-Small Cell Lung Cancer (first line) (PC0137-000)", https://www.cadth.ca/tagrisso-non-small-cell-lung-cancer-first-line-details |
| osimertinib | NICE | 2020 | "Osimertinib for untreated EGFR mutation-positive non-small-cell lung cancer - Technology appraisal guidance", www.nice.org.uk/guidance/ta621 |
| osimertinib | PBAC | 2019 | "Product Summary Document – July 2019 PBAC Meeting", https://www.pbs.gov.au/industry/listing/elements/pbac-meetings/psd/2019-07/files/osimertinib-psd-july-2019.pdf |
| osimertinib | ZIN | 2018 | "Pakketadvies osimertinib (Tagrisso®)", https://www.zorginstituutnederland.nl/publicaties/adviezen/2018/11/07/pakketadvies-sluisgeneesmiddel-osimertinib-tagrisso-bij-de-eerstelijnsbehandeling-van-patienten-met-gevorderde-of-gemetastaseerde-niet-kleincellige-longkanker-nsclc-met-activerende-egfr-mutaties |
| palbociclib | CADTH | 2016 | "Ibrance for Advanced Breast Cancer Resubmission (PC0093-000)", https://www.cadth.ca/ibrance-advanced-breast-cancer-resubmission-details |
| palbociclib | CADTH | 2019 | "Ibrance (with Faslodex) for Advanced or Metastatic Breast Cancer (PC0150-000)", https://www.cadth.ca/ibrance-faslodex-advanced-or-metastatic-breast-cancer-details |
| palbociclib | NICE | 2017 | "Palbociclib with an aromatase inhibitor for previously untreated, hormone receptor-positive, HER2-negative, locally advanced or metastatic breast cancer - Technology appraisal guidance", www.nice.org.uk/guidance/ta495 |
| palbociclib | NICE | 2020 | "Palbociclib with fulvestrant for treating hormone receptor-positive, HER2-negative, advanced breast cancer - Technology appraisal guidance", www.nice.org.uk/guidance/ta619 |
| palbociclib | PBAC | 2017 | "Public Summary Document – March 2017 PBAC Meeting", https://www.pbs.gov.au/industry/listing/elements/pbac-meetings/psd/2017-03/files/palbociclib-psd-march-2017.pdf |
| palbociclib | ZIN | 2017 | “Pakketadvies palbociclib (Ibrance®)”, https://www.zorginstituutnederland.nl/publicaties/adviezen/2017/04/11/pakketadvies-palbociclib |
| ribociclib | CADTH | 2018 | "Kisqali for Metastatic Breast Cancer (PC0112-000)", https://www.cadth.ca/kisqali-metastatic-breast-cancer-details |
| ribociclib | NICE | 2017 | "Ribociclib with an aromatase inhibitor for previously untreated, hormone receptorpositive, HER2-negative, locally advanced or metastatic breast cancer - Technology appraisal guidance", www.nice.org.uk/guidance/ta496 |
| ribociclib | PBAC | 2017 | "Public Summary Document - July 2017 PBAC meeting", https://www.pbs.gov.au/industry/listing/elements/pbac-meetings/psd/2017-07/files/ribociclib-psd-july-2017.pdf |
| ribociclib | ZIN | 2017 | “Pakketadvies ribociclib (Kisqali®)”, https://www.zorginstituutnederland.nl/publicaties/adviezen/2017/12/21/pakketadvies-ribociclib-kisqali-bij-gemetastaseerde-borstkanker |
| tisagenlecleucel ALL | NICE | 2018 | "Tisagenlecleucel for treating relapsed or refractory B-cell acute lymphoblastic leukaemia in people aged up to 25 years - Technology appraisal guidance", www.nice.org.uk/guidance/ta554 |
| tisagenlecleucel ALL | MSAC | 2019 | "Public Summary Document - Application No. 1519 – Tisagenlecleucel (CTL019) for treatment of refractory CD19-positive leukaemia and lymphoma", http://www.msac.gov.au/internet/msac/publishing.nsf/Content/1519-public |
| tisagenlecleucel ALL | ZIN | 2018 | "Pakketadvies sluisgeneesmiddel tisagenlecleucel (Kymriah®)", https://www.zorginstituutnederland.nl/publicaties/adviezen/2018/12/18/pakketadvies-sluisgeneesmiddel-tisagenlecleucel-kymriah-voor-de-behandeling-van-b-cel-acute-lymfatische-leukemie-b-cel-all-bij-kinderen-en-jongvolwassenen-tot-25-jaar |
| tisagenlecleucel DLBCL / ALL | CADTH | 2019 | "Tisagenlecleucel (Kymriah) for Pediatric Acute Lymphoblastic Leukemia and Diffuse Large B-Cell Lymphoma", https://www.cadth.ca/tisagenlecleucel-kymriah-pediatric-acute-lymphoblastic-leukemia-and-diffuse-large-b-cell-lymphoma |
| tisagenlecleucel DLBCL | NICE | 2019 | "Tisagenlecleucel for treating relapsed or refractory diffuse large B-cell lymphoma after 2 or more systemic therapies - Technology appraisal guidance", www.nice.org.uk/guidance/ta567 |
| tisagenlecleucel DLBCL | MSAC | 2019 | "Public Summary Document - Application No. 1519.1 – Tisagenlecleucel (CTL019) for treatment of relapsed or refractory diffuse large B-cell lymphoma (DLBCL)", http://www.msac.gov.au/internet/msac/publishing.nsf/Content/1519.1-public |
| tisagenlecleucel DLBCL | ZIN | 2019 | "Pakketadvies tisagenlecleucel (Kymriah®)", https://www.zorginstituutnederland.nl/publicaties/adviezen/2019/03/07/pakketadvies-sluisgeneesmiddel-tisagenlecleucel-kymriah |
| venetoclax & retuximab | CADTH | 2019 | "Venclexta in combo Rituximab for Chronic Lymphocytic Leukemia (PC0162-000)", https://www.cadth.ca/venclexta-combo-rituximab-chronic-lymphocytic-leukemia-details |
| venetoclax & retuximab | NICE | 2019 | "Venetoclax with rituximab for previously treated chronic lymphocytic leukaemia - Technology appraisal guidance", www.nice.org.uk/guidance/ta561 |
| venetoclax & retuximab | PBAC | 2018 | "Public Summary Document – November 2018 PBAC Meeting", https://www.pbs.gov.au/industry/listing/elements/pbac-meetings/psd/2018-11/files/venetoclax-psd-november-2018.pdf |
| venetoclax & retuximab | ZIN | 2019 | "Pakketadvies venetoclax (Venclyxto®)", https://www.zorginstituutnederland.nl/publicaties/adviezen/2019/05/08/pakketadvies-venetoclax-venclyxto-in-combinatie-met-rituximab |

ZIN = Zorginstituut Nederland, NICE = National Institute for Health and Care Excellence, CADTH = Canadian Agency for Drugs and Technologies in Health, PBAC = Pharmaceutical Benefit scheme and medical services Advisory, MSAC = Medical Services Advisory Committee

**S2 Table. References to the included HTA-reports related to the cases on orphan pharmaceuticals (n=21)**

| **Active ingredient /generic name (indication)** | **HTA organisation** | **Publication date** | **Title, URL** |
| --- | --- | --- | --- |
| eculizumab (PNH) | PBAC | July 2008 | “Public Summary Document July 2008 PBAC Meeting”, https://www.pbs.gov.au/industry/listing/elements/pbac-meetings/psd/2008-07/Eculizumab_Final_PSD_Alexion_Pharmaceuticals_Inc.pdf |
| eculizumab (PNH) | PBAC | March 2009 | “Public Summary Document March 2009 PBAC Meeting”, https://www.pbs.gov.au/industry/listing/elements/pbac-meetings/psd/2009-03/Eculizumab_Alexion_Pharmaceuticals_PSD_7-3_2009-03_Final.pdf |
| eculizumab (PNH) | CADTH | February 2010 | “Common drug review - CEDAC Final Recommendation Eculizumab New Indication:: Paroxysmal Nocturnal Hemoglobinuria”, https://www.cadth.ca/media/cdr/complete/cdr_complete_Soliris_February_18_2010.pdf |
| eculizumab (PNH) | PBAC | July 2010 | ”Public Summary Document July 2010 PBAC Meeting”, https://www.pbs.gov.au/industry/listing/elements/pbac-meetings/psd/2010-07/Eculizumab_SOLIRIS_Alexion.pdf |
| eculizumab (PNH) | ZIN | May 2016 | “Pakketadvies eculizumab (Soliris®) bij behandeling van PNH-patiënten”, https://www.zorginstituutnederland.nl/publicaties/adviezen/2016/05/13/pakketadvies-eculizumab-soliris-bij-behandeling-van-pnh-patienten |
| eculizumab (PNH) | ZIN | June 2017 | “Pakketadvies eculizumab (Soliris®) bij behandeling van PNH-patiënten”, https://www.zorginstituutnederland.nl/publicaties/adviezen/2017/06/09/pakketadvies-eculizumab-soliris-bij-pnh---herbeoordeling |
| eculizumab (aHus) | PBAC | March 2013 | “Public Summary Document March 2013 PBAC Meeting”, https://www.pbs.gov.au/industry/listing/elements/pbac-meetings/psd/2013-03/eculizumab-psd-03-2013.pdf |
| eculizumab (aHus) | CADTH | July 2013 | “Common drug review - CDEC Final Recommendation Eculizumab New Indication: Atypical Hemolytic Uremic Syndrome”, https://www.cadth.ca/sites/default/files/cdr/complete/cdr_complete_Soliris-aHUS_July-23-13.pdf |
| eculizumab (aHus) | PBAC | March 2014 | “Public Summary Document March 2014 PBAC Meeting”, https://www.pbs.gov.au/industry/listing/elements/pbac-meetings/psd/2014-03/eculizumab-psd-03-2014.pdf |
| eculizumab (aHus) | PBAC | August 2014 | “Public Summary Document July 2014 PBAC Meeting”, https://www.pbs.gov.au/industry/listing/elements/pbac-meetings/psd/2014-08/eculizumab-psd-07-2014.pdf |
| eculizumab (aHus) | NICE | January 2015 | “Eculizumab for treating atypical haemolytic uraemic syndrome - Highly specialised technologies guidance”, https://www.nice.org.uk/guidance/hst1 |
| eculizumab (aHus) | CADTH | May 2015 | “Common drug review - CDEC Record of Advice Eculizumab New Indication: Atypical Hemolytic Uremic Syndrome”, https://www.cadth.ca/sites/default/files/cdr/advice/cdr-advice-Soliris-aHUS-June-2-2015.pdf |
| eculizumab (aHus) | ZIN | November 2016 | “Pakketadvies eculizumab (Soliris®) bij behandeling van aHUS-patiënten”, https://www.zorginstituutnederland.nl/publicaties/adviezen/2016/11/21/pakketadvies-eculizumab-soliris-bij-behandeling-van-ahus-patienten |
| Lumacaftor/ ivacaftor (CF) | PBAC | March 2016 | “Public Summary Document – March 2016 PBAC Meeting”, https://www.pbs.gov.au/industry/listing/elements/pbac-meetings/psd/2016-03/files/lumacaftor-ivacaftor-psd-march-2016.pdf |
| Lumacaftor/ ivacaftor (CF) | ZIN | May 2016 | “GVS rapport 16/08 lumacaftor/ivacaftor (Orkambi®)”, https://www.zorginstituutnederland.nl/publicaties/adviezen/2016/05/13/gvs-advies-lumacaftor-ivacaftor-orkambi-bij-cystische-fibrose-cf-bij-patienten-van-12-jaar-en-ouder-die-homozygoot-zijn-voor-de-f508del-mutatie-in-het-cftr-gen |
| Lumacaftor/ ivacaftor (CF) | NICE | July 2016 | “Lumacaftor–ivacaftor for treating cystic fibrosis homozygous for the F508del mutation - Technology appraisal guidance”, www.nice.org.uk/guidance/ta398 |
| Lumacaftor/ ivacaftor (CF) | CADTH | October 2016 | “Common drug review – CADTH Canadian Drug Expert Committee Final Recommendation Lumacaftor / Ivacaftor Indication: Cystic Fibrosis, F508del-CFTR mutation”, https://www.cadth.ca/sites/default/files/cdr/complete/SR0471_complete_Orkambi-Oct-28-16.pdf |
| Lumacaftor/ ivacaftor (CF) | PBAC | November 2016 | “Public Summary Document – November 2016 PBAC Meeting”, https://www.pbs.gov.au/industry/listing/elements/pbac-meetings/psd/2016-11/files/lumacaftor-ivacaftor-psd-november-2016.pdf |
| Lumacaftor/ ivacaftor (CF) | ZIN | December 2016 | “Herbeoordeling lumacaftor/ivacaftor (Orkambi)” https://www.zorginstituutnederland.nl/publicaties/adviezen/2016/12/15/gvs-advies-lumacaftor-ivacaftor-orkambi-bij-cystische-fibrose-cf-bij-patienten-van-12-jaar-en-ouder-die-homozygoot-zijn-voor-de-f508del-mutatie-in-het-cftr-gen-herbeoordeling |
| Lumacaftor/ ivacaftor (CF) | PBAC | July 2017 | “Public Summary Document – July 2017 PBAC Meeting”, https://www.pbs.gov.au/industry/listing/elements/pbac-meetings/psd/2017-07/files/lumacaftor-ivacaftor-psd-july-2017.pdf |
| Lumacaftor/ ivacaftor (CF) | PBAC | July 2018 | “Public Summary Document – July 2018 PBAC Meeting”, https://www.pbs.gov.au/industry/listing/elements/pbac-meetings/psd/2018-07/files/lumacaftor-with-ivacaftor-age-12-over-psd-july-2018.docx.pdf |

ZIN = Zorginstituut Nederland, NICE = National Institute for Health and Care Excellence, CADTH = Canadian Agency for Drugs and Technologies in Health, PBAC = Pharmaceutical Benefit scheme and medical services Advisory
